# Is the Indian health system resilient? Lessons from COVID-19

**DOI:** 10.7189/jogh.12.03041

**Published:** 2022-07-06

**Authors:** Vikas Sheel, Tarannum Ahmed, Neha Dumka, Erin Hannah, Vishal Chauhan, Atul Kotwal

**Affiliations:** 1 National Health Mission, Ministry of Health and Family Welfare, New Delhi, India; 2 Knowledge Management Division, National Health Systems Resource Centre, Ministry of Health and Family Welfare, New Delhi, India; 3 National Health Mission, Ministry of Health and Family Welfare, New Delhi, India; 4 National Health Systems Resource Centre, Ministry of Health and Family Welfare, New Delhi, India

**Figure:**
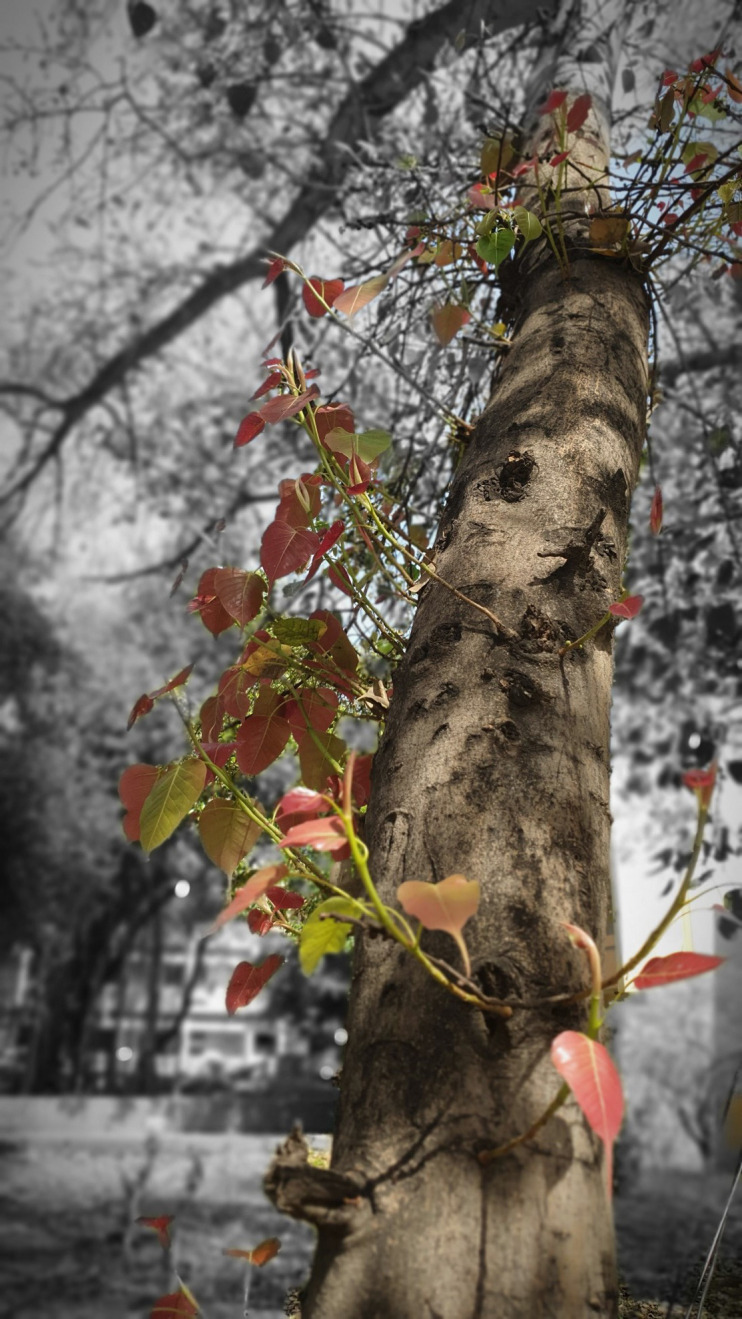
Photo: Resilience (from the collection of Dr Neha Dumka, used with permission).

The importance of health system resilience has been brought to the forefront by the COVID-19 pandemic. The dramatic loss of human life, massive economic and social disruption, and collapse of health care services worldwide demonstrate the consequences of an unprecedented disaster on health systems that are not sufficiently built to withstand health shocks [1]. The Indian health system faced multiple challenges concerning, but not limited to, governance, infrastructure, and manpower. It witnessed significant losses in the form of a raging death toll as a result of an increasing patient load [2]. However, it also administered pre-emptive and proactive steps to provide a coherent response to the pandemic. It utilized the extenuating circumstances as an opportunity to understand key limitations and repurpose and strengthen the health system [2]. In other words, it showed resilience.

This viewpoint puts forth the Indian health system’s resilience by analysing its “whole-of-government” and “whole-of-society” approach from lessons learnt from COVID-19. It does so by utilizing a proposed framework of resilient health system [3] **(****Figure 1**) and the proposed Harvard resilience index based on it **(**Appendix S1 of the **Online Supplementary Document**) [4]. The five elements derived from the proposed framework [3], ie, aware, integrated, diverse, self-regulating, and adaptive, will be used to characterize the Indian health system’s resilience.

**Figure 1:**
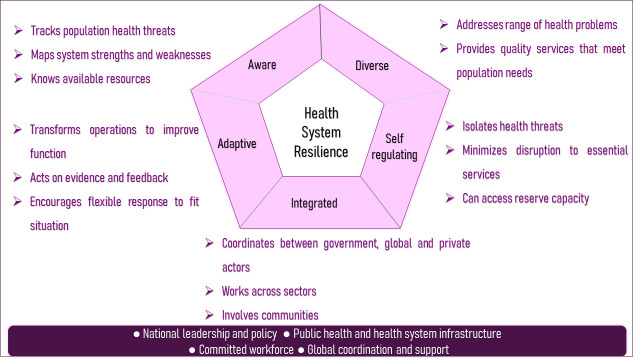
Resilient health system [3].

Although this framework is ideally adopted before the event for dynamic performance, this viewpoint evaluates these features based on the efforts made by the Union Ministry of Health and Family Welfare (MoHFW) in collaboration with diverse stakeholders during the pandemic. Apart from demonstrating the Indian health system’s resilience, this has been done to indicate areas for action towards the long-standing functions of health system strengthening for policymakers and relevant stakeholders. Additionally, we hope India’s experience will demonstrate how resilience can be built in the low- and middle-income countries (LMIC) context. The following sections will discuss each element in detail.

## RESILIENNCE MATRIX SYSTEM: IN THE INDIAN CONTEXT

First, resilient health systems are aware. They have an up-to-date record of human, physical, and information sources that shed light on the health system assets and weaknesses [3]. The empowered group 4 constituted by the Government of India (GoI) launched an online data pool of state-wise human resources (HR) and volunteers that helped authorities prepare contingency plans in the functional areas [2,5]. COVID-19 facility preparedness was assessed through the “COVID India portal” previously known as the “Special Surveillance System (S-3)” which constituted real-time data on infrastructure, equipment, consumables and community surveillance [2]. Community surveillance was further bolstered by the “Aarogya Setu” application which enabled the early identification and prevention of potential risk of infection through contact tracing and self- assessment features, as well as the identification of potential hotspots to control and mitigate the spread of COVID-19 [2]. Another application called “ITIHAS” analysed information for latitude and longitude scores to locate all COVID-19 patients and reflected the status of infection in an area [2]. The continuously refined and upgraded epidemiological surveillance systems not only informed the current status of COVID-19, but also alerted the health authorities of an impending health threat. This facilitated predictive modelling and evidence-based decision-making by decision makers in key sectors.

In addition to distribution of assets, efforts were made to analyse health service utilization trends through the COVID Facility Application that recorded facility-level entry about patients (admission, treatment and outcome details) and transferred all data to the COVID India portal for further action [2]. The stringent information system was complemented by a comprehensive communications strategy adopted by the GoI by implementing several effective initiatives (early communication strategy, early campaign of COVID-19 dos & don’ts – pre-caller tunes, lockdown communication, mental health communication, campaigns on stigma, COVID-19 appropriate behaviours, and panel discussion on television) [2] to simulate the logistics of India’s response to the pandemic. However, community resistance and non-compliance fuelled by misinformation challenged health system response. This was indicated during the first and second wave of COVID-19 in India where unfortunate incidents of violence against the health care workers in community settings, hospitals and caregiving institutions were reported across the country. To learn from such episodes, we need to deeply understand what community engagement is and how it works. To avert similar crises, systematic changes are needed in varying contexts.

The second element of resilience pertains to health systems being diverse and having the capacity to effectively respond to a range of health care challenges, adequately finance them, and circumvent financial adversities [4]. Primary health care is an important example of a diverse platform. Based on previous experiences, India promptly anticipated a disruption in the delivery of essential health services and issued a detailed guidance note to ensure their continuation [6]. Nevertheless, some challenges remain; the linkages between the services under primary health care and the financial provisions under National Health Protection Scheme need to be bolstered.

Additionally, the “eSanjeevani” portal was launched as an online OPD service to maintain the continuity of health care services for patients at their homes [2]. This systematic approach served as an alternative to diversify access to essential non-COVID services, enhance people’s trust in the system, minimize mortality and morbidity from other conditions and enabled rapid detection of potential health threats [2]. Yet, various factors contributing to digital divide that constrain access need to be addressed.

Another essential resilience measure – financing – was provided in the form of a strong financial impetus from the National Health Mission (NHM). Emergency COVID-19 Response Plan (ECRP) I and II aided infrastructure, logistics and technological reinforcements to enhance health system preparedness. The Fifteenth Finance Commission (FC-XV) recommended grants for specific health sector components in the amount of Rs. 70 051 crores to strengthen the existing infrastructural system at the grass-root level. Further, the Prime Minister – Ayushman Bharat Health Infrastructure Mission (PM-ABHIM) committed Rs. 64 120 crores over a period of five years to fill critical gaps in health care provisioning [2]. Additional loans were enabled from lending organizations and mechanisms were created for voluntary contributions [7,8]. Reallocation of available funds was facilitated to address urgent needs, price capping was enabled in the private sector to protect vulnerable families from financial constraints, and insurance schemes such as the PMJAY were reformed to include COVID-19-related health issues. Despite the price capping, service affordability, especially in the private sector, was a challenge. The situation draws attention to the need for regulatory reforms in the private sector to enable ease of access and amend them as required during crises. Nonetheless, these timely measures fostered a financially secure environment for the people and encouraged them to seek care in health facilities, which helped the government contain an outbreak of global magnitude.

Third, health systems are self-regulating: they can isolate threats, maintain core function, and minimize disruption to the provision of essential health services during crises [3]. The MoHFW established agreements with private providers, not-for-profit, and for-profit institutions, including (among others) the World Health Organization (WHO), World Bank, All India Institute of Medical Sciences (AIIMS) and Indian Medical Association (IMA), with the aim to expand service provision and promote synergistic actions as an example of smart dependency [2]. Another factor that determines the ability to self-regulate is the existence of an alternate service delivery database for affected and unaffected populations, to lower redundant reinventions [4]. The GoI routinely updated existing service delivery models, launched, and implemented multiple research initiatives in consultation with experts. Among the approximately 200 COVID-19-related technologies for tracking, testing, surveillance, treatment, and prevention, only those that were deemed effective were scaled up and implemented by the MoHFW [2]. These measures aided in the construction of a steady platform for service delivery with an infusion of speedy variables such as quarantine/isolation/management units to strengthen crisis response [9-11]. Going forward, strengthening intersectoral convergence for effective coverage and penetration of health services within relevant non-health departments is required. Additionally, the ability to self-regulate and adapt by various actors must be capitalized to strengthen the health systems continually.

The fourth element refers to the quality of integration; it assembles diverse ideas, actors and groups to formulate solutions and commence action [3]. The MoHFW took protective steps as early as January 2020 to contain the spread of the virus by continuously monitoring the situation in collaboration with the WHO [2,12]. Defined agreements and Standard Operating Procedures (SOPs) were developed to clarify the roles of health facilities at all levels to avoid confusion and service delay [2]. Joint planning sessions were routinely conducted to rehearse preparedness plans and promote intersectoral teamwork [2]. Capacity building of public health staff of all cadre was conducted by multiple virtual training sessions delivered by the MoHFW, AIIMS, and WHO India [2]. A new cadre of health care workers, the Community Health Officer (CHO), was introduced to aid the provision of Comprehensive Primary Healthcare (CPHC) at the Ayushman Bharat Health and Wellness Centers (AB-HWCs). The ASHAs, Self-help groups, local volunteers and community-based NGOs promptly addressed community needs through mobilization and engagement activities [2]. In Karnataka and Kerala, a Panchayat level Task Force with frontline workers and PRI members as leaders was created at the village level to formulate COVID-19 management plans [2]. This and other such initiatives provided a platform for dialogue with the leaders which instilled trust in the population, facilitating community participation and effective service delivery.

Another prominent example of integration from the Indian health system was the inclusion of experts from diverse fields of sociology, anthropology, and health care-related disciplines to understand key determinants of the emergency response. Since February 2020, the Indian Council of Medical Research (ICMR) has assiduously worked towards the development of research and technological innovations to control the virus [2]. A firm policy momentum for the development of a “One Health” strategy [13] was also palpable when the 2021 union budget allocated a specific portion for its effective implementation. Henceforth, investments have been made to develop core capacity to deliver the One Health approach to prevent, detect, and respond to infectious disease outbreaks in animals and humans [14].

Given the presence of multiple stakeholders and different programme divisions, duplication of efforts and lack of communication have also been barriers towards the nation's goal of effective integration. Realizing that effective governance and strong intersectoral coordination are critical to a well-functioning health system, India should continue to adopt a holistic approach to take all stakeholders together while implementing National Health programmes with a citizen centric approach.

Lastly, resilience is about adaptability – the ability to transform and improve both short-term and long-term functionality in the face of adversity. For that, agreements on delegation of authority and funding during crises have been formulated by the GoI through the constitution of 11 Empowered Groups under the 2005 Disaster Management Act 2005 to plan and initiate action during emergencies [2]. Subsequently, the MoHFW made formal provisions for flexible spending of funds during an emergency. For example, India’s ECRP is a 100% Centrally Sponsored Scheme financed with the support of World Bank and other financial institutions (ECRP I – Rs 15 000 crores and ECRP II – Rs15 000 crores) for COVID-19 and future outbreaks [2]. Moreover, district and local health teams are trained in a manner to interpret real-time data and take quick evidence-informed decisions during an emergency [2]. The GoI’s mechanisms and capacity for tracking progress during a crisis have been well-demonstrated. The open access information systems and self-evaluative reports published by the MoHFW validates India’s efforts towards the goal of “evaluate to improve”. The narrative review titled “Chasing the Virus Volume I” documents India’s response to the COVID-19 pandemic from January 2020 to November 2020. Volume II will document the initiatives taken December 2020 onward. Both the reports on past responses provide essential feedback for further adaptation.

## CONCLUSION

In our view, the Indian health system has imbibed all the essential elements that make a health system resilient. Despite the COVID-19 pandemic reverberating through communities and economies and posing formidable challenges, the Indian health system constructively responded to it through evidence and databased scientific planning, strategic infusion of funds and effective implementation. Like many other health systems worldwide, it witnessed significant setbacks in the face of the pandemic but showed resilience. The MoHFW made continuous and concerted efforts and to make those efforts even more worthwhile, it made long-term changes to the public health system. The recent initiatives [15] are testimony to the GoI’s resilience and proactive response to the pandemic and commitment to improved health care provision even in the time of adversity. We hope this viewpoint helps inform India’s resilience to COVID-19 and demonstrates how resilience can be built in LMICs and the entire world even during a crisis.

## Additional material


Online Supplementary Document

## References

[1] Kieny MP Evans DB Schmets G Kadandale S Health-system resilience: reflections on the Ebola crisis in western Africa. Bull World Health Organ. 2014;92:850. 10.2471/BLT.14.149278 25552765 PMC4264399

[2] Chasing the Virus - A Public Health Response to the COVID-19 PandemicAvailable: https://nhsrcindia.org/sites/default/files/2021-07/Chasing_the_Virus_A_Public_Health_Response_to_the_COVID-19_Pandemic_02032021_1.pdf. Accessed: 15 February 2022.

[3] Kruk ME Myers M Varpilah ST Dahn BT What is a resilient health system? Lessons from Ebola. Lancet. 2015;385:1910-2. 10.1016/S0140-6736(15)60755-3 25987159

[4] Kruk ME Ling EJ Bitton A Cammett M Cavanaugh K Chopra M Building resilient health systems: a proposal for a resilience index. BMJ. 2017;357:j2323. 10.1136/bmj.j2323 28536191

[5] Order Dt MHA. 29.3.2020 on Disaster Management Act 2005.pdf. Available: https://dst.gov.in/sites/default/files/MHA%20Order%20Dt.%2029.3.2020%20on%20%20Disaster%20Management%20Act%202005.pdf. Accessed: 15 February 2022.

[6] Guidance Note on Provision of essential RMNCAHN Services. Available: https://www.mohfw.gov.in/pdf/GuidanceNoteonProvisionofessentialRMNCAHNServices24052020.pdf. Accessed: 17 February 2022.

[7] NHM Finance - FMG. National Health Mission. Available: https://nhm.gov.in/index1.php?lang=1&level=1&sublinkid=1039&lid=166. Accessed: 6 January 2022.

[8] PM CARES Fund - PM’s Citizen Assistance & Relief in Emergency Situations FundAvailable: https://www.pmcares.gov.in. Accessed: 6 January 2022.

[9] Loss of Resilience, Crisis, and Institutional Change: Lessons from an Intensive Agricultural System in Southeastern Australia. Ecosystems (N Y). 2006;9:865-78. 10.1007/s10021-006-0017-1

[10] Carpenter SR Ludwig D Brock WA Management of Eutrophication for Lakes Subject to Potentially Irreversible Change. Ecol Appl. 1999;9:751-77. 10.1890/1051-0761(1999)009[0751:MOEFLS]2.0.CO;2

[11] Anderies JM Janssen MA Walker BH Grazing Management, Resilience, and the Dynamics of a Fire-driven Rangeland System. Ecosystems (N Y). 2002;5:23-44. 10.1007/s10021-001-0053-9

[12] DGS Order dated 04,2020. Available: https://onedrive.live.com/?authkey=%21Aut6ewfe2AvQhNQ&cid=061DC39C09D4F695&id=61DC39C09D4F695%2127115&parId=61DC39C09D4F695%2127095&o=OneUp. Accessed: 6 January 2022.

[13] Asaaga FA Young JC Oommen MA Chandarana R August J Joshi J Operationalizing the “One Health” approach in India: facilitators of and barriers to effective cross-sector convergence for zoonoses prevention and control. BMC Public Health. 2021;21:1517. 10.1186/s12889-021-11545-7 34362321 PMC8342985

[14] Budget SpeechAvailable: https://www.indiabudget.gov.in/doc/budget_speech.pdf. Accessed: 15 February 2022.

[15] Pradhan Mantri - Ayushman Bharat Health Infrastructure Mission | National Health Systems Resource Centre. Available: https://nhsrcindia.org/pradhan-mantri-aatmanirbhar-swasthya-bharat-pm-asby. Accessed: 6 Jan 2022.

