# Supplementary material for: Is the Indian health system resilient? Lessons from COVID-19
Source: J Glob Health. 2022 Jul 6;12:03041. doi: 10.7189/jogh.12.03041 (PMC9253556; doi:10.7189/jogh.12.03041)
Supplement: Online Supplementary Document [file jogh-12-03041-s001.pdf]

Appendix 1: Resilience Index[4]

Source: Kruk ME et al. Building resilient health systems: a proposal for a resilience index.

Characteristics	Aims	Measures	Rationale
**Aware**	Know health system capacity	1. Distribution of health system assets and weaknesses	Real time geo-registry of HWs, supplies, and facilities (including NGOs and private operations) can realistically gauge available national capacities
		2. Health service utilisation trends	Routine health monitoring helps system detect service fluctuations and accurate assessments of crisis impact, and rate of return to baseline after a shock
	Know risks and population	3. Presence of active epidemiologic surveillance system	Routine surveillance is necessary to detect disease threats and trigger mitigation mechanisms
		4. Functioning civil registration and vital statistics system	Basic knowledge of population demographics is important for estimating health threats and trends, and understand crisis impact
	Communicate	5. List of decision makers in key sectors	Point persons across sectors must be immediately accessible for communication, decision making, and sounding alarms
		6. Breadth of functioning communication channels	Communities must be able to notify and sound alarms—this requires an environment of free speech and freedom of press, and functioning, open platforms for timely communication (hotlines, community committees, social media)

**Diverse**	Effectively respond to range of health needs	7. Scope of health services available in primary care	Including services that respond to population health needs and expectations in basic primary care package will promote routine health system utilisation and confidence in the health system
		8. Quality of care for sentinel conditions in basic package	Health outcomes, healthcare utilisation during crisis, and trust in health authorities require competent and respectful care
	Adequately finance health systems; prevent financial harm	9. Financing of healthcare: adequacy of government health expenditure and financial protection	Total health system funding must be sufficient to support functioning services; financing systems should aim to reduce catastrophic and impoverishing health spending
**Self-regulating**	Isolate threat and maintain core function	10. Isolate threat and maintain core function	Establishing agreement about roles for private providers—not for profit and for profit—in crisis expands service provision in emergencies and may promote collaboration in times of calm
		11. Database of service delivery alternatives for affected and unaffected populations	A routinely updated global, open access library of service delivery models tested and deemed effective in past crises promotes inter-country learning and lowers redundant reinvention and perpetuation of failed ideas
	Leverage outside capacity	12. Collaboration agreements with regional and global actors	Agreements on nature of collaboration (timing, type of support, roles or responsibilities) during emergencies is a form of smart dependency and contributes to a faster, more effective response

**Integrated**	Coordinate with non-health actors (education, transport, police, media, private enterprise)	13. Existence of a national emergency coordination system and leaders	Ready coordination systems encourage fast decision making and implementation, curbing potential effects of emergencies
		14. Frequency of joint planning sessions and drills	Rehearsal of preparedness plans and regular collaboration establishes norms of intersectoral teamwork
	Engage citizens and communities to build trust	15. Process for development of a One Health strategy	Acknowledging human ties to the environment and other species encourages an inclusive understanding of public health vulnerabilities
		16. Index of Ministry of Health and government responsiveness to community need	Quick action in responding to community needs can foster trust and promote containment of health shock
		17. Population trust in health system	Trust in government and the health system is essential to effective service delivery and for acceptance of government messages in crises—this is true in government run and mixed provider health systems
		18. Platforms for dialogue with community leaders	Regular input about health system functioning from citizens will improve emergency planning and establish communication channels for routine and emergency needs

		19. In-country social scientists with experience working with health departments	Tapping experts in sociology, anthropology, and related disciplines strengthens understanding of key social structures in crisis response, local health determinants and the local appropriateness and acceptability of interventions
	Link healthcare provision to public health	20. Availability of district health staff with public health training	Public health staff serve to promote public health practices and act as sentinels for potential outbreaks connecting local clinics to surveillance and monitoring system
	Coordinate primary and referral care	21. Agreement on roles and referral protocols for facilities	Defined agreements on the role of primary and referral facilities reduces confusion and service delay, and streamlines service delivery for patients
**Adaptive**	Shift resources to meet need	22. Formal provisions to reallocate funds in emergency	Flexible spending of funds—national and international—speeds up and better targets emergency response in fast changing situations
	Promote rapid local decision making	23. Management capacity of district or local health teams	For decentralised responses, local health teams must be able to interpret local data and local leaders must be able to make quick and sound operational decisions
		24. Agreements on delegation of authority and funding in crises	Pre-crisis agreements permitting local decision making in crisis with sufficient support hasten response time to evolving challenges

	Evaluate to improve	25. Mechanisms for, and capacity to, track progress and evaluate health system performance in crisis and in times of calm	Rigorous monitoring during crisis and independent evaluation post-crisis permits course correction and points to needed reforms. National capacity for data use and, more broadly, a culture of open inquiry and evaluation needs to be built in times of calm to deliver during a crisis.
